# Mild Chronic Kidney Disease Associated with Low Bone Formation and Decrease in Phosphate Transporters and Signaling Pathways Gene Expression

**DOI:** 10.3390/ijms24087270

**Published:** 2023-04-14

**Authors:** Evdokia Bogdanova, Airat Sadykov, Galina Ivanova, Irina Zubina, Olga Beresneva, Natalia Semenova, Olga Galkina, Marina Parastaeva, Vladimir Sharoyko, Vladimir Dobronravov

**Affiliations:** 1 Research Institute of Nephrology, Pavlov University, 197022 Saint Petersburg, Russia; 2 Raisa Gorbacheva Memorial Research Institute for Pediatric Oncology, Hematology and Transplantation Pavlov University, 197022 Saint Petersburg, Russia; 3 Laboratory of Cardiovascular and Lymphatic Systems, Physiology Pavlov Institute of Physiology, 199034 Saint Petersburg, Russia; 4 Research Department of Pathomorphology, Almazov National Medical Research Center, 197341 Saint Petersburg, Russia; 5 Department of General and Bioorganic Chemistry, Pavlov University, 197022 Saint Petersburg, Russia

**Keywords:** chronic kidney disease, bone remodeling, static bone histomorphometry, inorganic phosphate transporters, intracellular signaling

## Abstract

The initial phases of molecular and cellular maladaptive bone responses in early chronic kidney disease (CKD) remain mostly unknown. We induced mild CKD in spontaneously hypertensive rats (SHR) by either causing arterial hypertension lasting six months (sham-operated rats, SO6) or in its’ combination with 3/4 nephrectomy lasting two and six months (Nx2 and Nx6, respectively). Sham-operated SHRs (SO2) and Wistar Kyoto rats (WKY2) with a two-month follow-up served as controls. Animals were fed standard chow containing 0.6% phosphate. Upon follow-up completion in each animal, we measured creatinine clearance, urine albumin-to-creatinine ratio, renal interstitial fibrosis, inorganic phosphate (Pi) exchange, intact parathyroid hormone (PTH), fibroblast growth factor 23 (FGF23), Klotho, Dickkopf-1, sclerostin, and assessed bone response by static histomorphometry and gene expression profiles. The mild CKD groups had no increase in renal Pi excretion, FGF23, or PTH levels. Serum Pi, Dickkopf-1, and sclerostin were higher in Nx6. A decrease in trabecular bone area and osteocyte number was obvious in SO6. Nx2 and Nx6 had additionally lower osteoblast numbers. The decline in eroded perimeter, a resorption index, was only apparent in Nx6. Significant downregulation of genes related to Pi transport, MAPK, WNT, and BMP signaling accompanied histological alterations in Nx2 and Nx6. We found an association between mild CKD and histological and molecular features suggesting lower bone turnover, which occurred at normal levels of systemic Pi-regulating factors.

## 1. Introduction

Inorganic phosphate (Pi) retention is a hallmark of progressive chronic kidney disease (CKD) [1]. Hyperphosphatemia, the indicator of a positive Pi balance, is a major predictor of adverse clinical events and a therapeutic target [2,3]. An increase in the level of circulating Pi typically develops at a significant decrease in the nephron’s number (i.e., to <30–40%) [1]. Phosphate *per se*, independent of calcium and calcitriol, leads to the development of parathyroid gland hyperplasia and secondary hyperparathyroidism [4]. A response of systemic endocrine factors (i.e., parathyroid hormone (PTH) and fibroblast growth factor 23 (FGF23)) counterbalances renal phosphate retention and delays hyperphosphatemia at earlier CKD stages [5,6,7,8,9]. Besides phosphate and calcium imbalance and its endocrine and paracrine environment, overt CKD is also associated with progressive alterations of the skeleton concurrently with myocardium remodeling and vascular calcification, known as chronic kidney disease—mineral and bone disorder (CKD-MBD) [3,8,9,10,11,12,13].

There is a direct correlation between the degree of chronic kidney injury and skeletal abnormalities [12], which becomes apparent in almost all patients with end-stage renal disease [14,15,16,17,18,19]. CKD-MBD results in a variety of bone phenotypes [2,7,8,10,11,12,13,15,16,17,18,19,20,21]. Among them, osteitis fibrosa (a high bone turnover), adynamic bone disorder (a low bone turnover), or their combinations are prevalent [2,8,10,11,12,13,15,16,17,18,19,20,21].

Most studies of CKD-MBD, however, were performed in animal or clinical models of overt CKD or end-stage renal disease [10,11,14,15,16,17,18]. Particularly, 5/6 nephrectomy or genetic models used in experimental CKD-MBD are characterized by at least a 50% decline in glomerular filtration rate, which corresponds to moderate-to-severe human CKD [6,7,8,10,11,13]. Notably, bone alterations in these models occurred against the background of hyperphosphatemia [7,8,11,13] and significant changes in the circulating levels of FGF23, PTH [7,10,12,13], Dickkopf-1, and sclerostin [6,8,13]. 

In the face of an apparent increase in PTH level, moderate-to-severe CKD is associated with high bone turnover mediated by canonical WNT (cWnt) up-regulation [2,7,8,9,10,15,16,18,19]. Contrariwise, low bone turnover is more prevalent in mild-to-moderate CKD and is associated with normal or suppressed PTH [2,8,12,13,18,19,20] and up-regulation of cWnt inhibitors (iWnt) [8,13]. A paucity of data on bone remodeling in early CKD [8,12,13] highlights an issue of the initial phases of CKD-MBD pathogenesis and related regulatory factors.

Studies of bone turnover and associated molecular events in mild CKD prior to the hyperphosphatemia and the increase in Pi-regulatory hormones are currently lacking. This knowledge, however, might be of importance for the development of strategies aiming to prevent the overt, mainly irreversible, consequences of CKD-MBD. In this study, we focused on the in vivo assessment of bone response at the level of bone histology and gene expression profile in the early stages of experimental CKD.

## 2. Results

### 2.1. Animal Models of Chronic Kidney Disease 

#### 2.1.1. Features of Mild Chronic Kidney Disease

All experimental groups (SO6, Nx2, Nx6) demonstrated a 6–12-fold increase in albuminuria with either normal (SO6) or decreased by 30% (Nx2, Nx6) creatinine clearance (CCr) compared to control (Table 1). Additionally, serum Klotho decline and a mild, but statistically significant, increase in interstitial fibrosis (IF) area were obvious in all experimental groups (Table 1). Albuminuria, serum Klotho, CCr, and the IF area in SO2 did not differ from normotensive control (WKY2) (Table 1).

#### 2.1.2. Phosphate and Its Regulators

The serum level of Pi did not increase in SO6, Nx2, and was higher in Nx6 compared to the control and other experimental groups (Table 1). Urinary Pi excretion, bone and kidney tissue phosphorus content, FGF23, and PTH levels had no significant differences between study groups (Table 1). Nx6 animals had significantly elevated serum Dickkopf-1 and sclerostin concentrations compared to SO6, and Nx2 animals have higher serum Dickkopf-1 compared to SO6 (Table 1).

#### 2.1.3. Static Bone Histomorphometry

**Figure 1:**
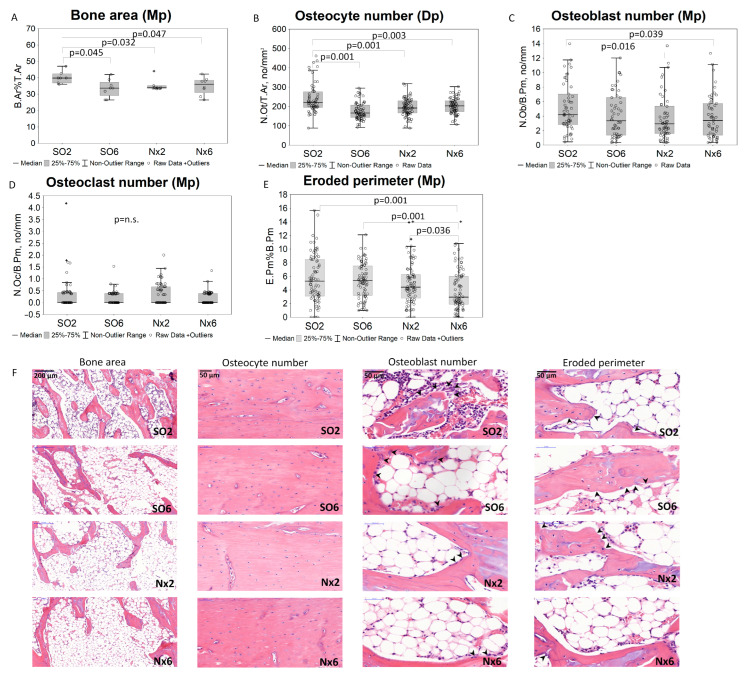
Bone turnover in mild CKD models—static bone histomorphometry: (**A**)—bone area (B.Ar%T.Ar; n = 8 for each group); (**B**)—osteocyte number in the diaphyseal (Dp) region (N.Ot/T.Ar, no/mm^2^; n(SO2) = 72, n(SO6) = 74, n(Nx2) = 73, n(Nx6) = 72); (**C**)—osteoblast number in the metaphyseal (Mp) region (N.Ob/B.Pm, no/mm; n = 80 for each group); (**D**)—osteoclast number (N.Oc/B.Pm, no/mm; n(SO2) = 74, n(SO6) = 76, n(Nx2) = 72, n(Nx6) = 77); (**E**)—eroded perimeter (E.Pm.%B.Pm; n = 80 in each group); non-significant differences between groups are not indicated; (**F**)—representative microphotographs for the bone static histomorphometry parameters in the control (SO2) and mild CKD (SO6, Nx2, and Nx6) groups (H&E; arrowheads point osteoblasts and eroded perimeter at the corresponded pictures); n.s.—not significant.

A subtle but statistically significant decrease in trabecular bone area (Figure 1A,F) and osteocyte number (Figure 1B,F) occurred in SO6 compared with SO2. Having more advanced kidney injury, the Nx2 and Nx6 groups additionally exhibited a lower osteoblast number (Figure 1C,F). The decline in eroded perimeter (Figure 1E,F) was also apparent in Nx6. Osteoclast number (Figure 1D) did not significantly differ between study groups.

### 2.2. Bone Gene Expression in Mild Chronic Kidney Disease Models

**Figure 2:**
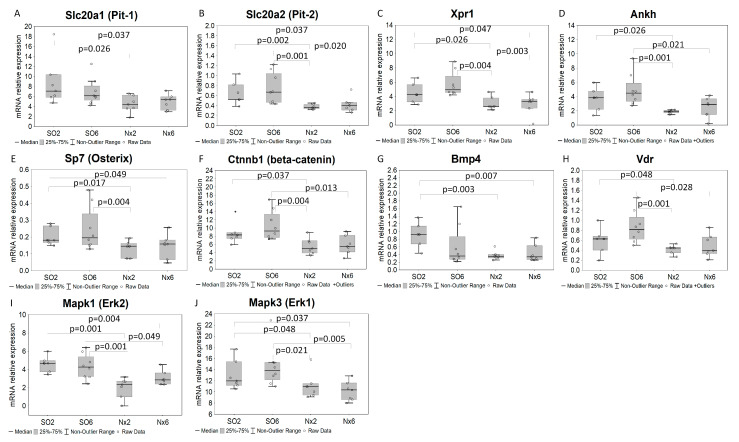
Bone turnover in mild CKD models—mRNA relative expression in bone: (**A**)—*Slc20a1*, solute carrier family 20 member 1 (Pit-1); (**B**)—*Slc20a2*, solute carrier family 20 member 2 (Pit2); (**C**)—*Xpr1*, xenotropic and polytropic retrovirus receptor 1; (**D**)—*Ankh*, ANKH pyrophosphate transport regulator; (**E**)—*Sp7*, transcription factor osterix; (**F**)—*Ctnnb1*, catenin beta 1; (**G**)—*Bmp4,* bone morphogenetic protein 4; (**H**)—*Vdr*, vitamin D receptor; (**I**)—*Mapk1*, mitogen-activated protein kinase 1 (Erk2); (**J**)—*Mapk3*, mitogen-activated protein kinase 3 (Erk1).

We found a significant downregulation of genes related to Pi transport (*Slc20a1*, *Slc20a2*, *Xpr1*, *Ankh*; Figure 2A–D) and cellular signaling (*Sp7*, *Ctnnb1*, *Bmp4*, *Vdr*, *Mapk1/3*, Figure 2E–G; *Fgfr2*; Table S2) in Nx2 and Nx6 groups vs. SO2 and SO6, but not in SO6 vs. SO2 (Figure S1). Bone expressions of *Dkk1*, *Sost*, *Kl, Fgf23*, *Lgr4*, *Tnfrsf11b*, *Tnfsf11*, *Cyp27b1*, *Sfrp2*, *Fzd2*, and *Wnt10b* (Table S2) had no significant differences between groups (Figure S1). The predicted interaction networks and their likely involvement in the biological processes of the differentially expressed genes’ products are shown in Figure S4.

### 2.3. Bone Immunohistochemistry in Mild Chronic Kidney Disease Models

**Figure 3:**
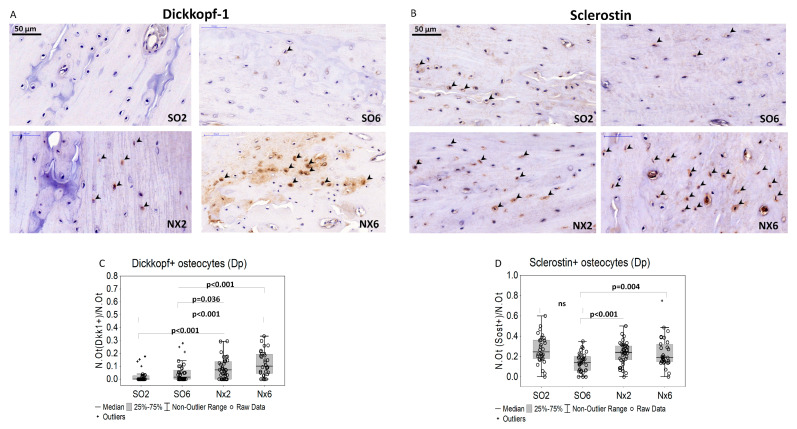
Representative microphotographs for bone Dickkopf-1 (**A**) and sclerostin (**B**) IHC staining in the distal diaphyseal region (black arrowheads—IHC-positive osteocytes); the ratio of dickkopf-1-positive ((**C**), N.Ot(Dkk1+)/N.Ot, n = 40 for each group) and sclerostin-positive osteocytes ((**D**), N.Ot(Sost+)/N.Ot, n = 40 for each group); ns—not significant.

Dickkopf-1-positive staining was found in osteocytes (Figure 3A), endotheliocytes, cartilage cells, and extracellular matrix within the cartilage zone, as well as in bone marrow (Figure S2). Sclerostin is expressed in osteocytes, endotheliocytes (Figure 3B), chondroblasts, and bone marrow cells (Figure S3). The proportion of Dickkopf-1-positive osteocytes in the diaphysis increased in Nx2 and Nx6 versus the SO2 group (control) and the SO6 group (mild CKD) (Figure 3C). The proportion of sclerostin-positive osteocytes in the diaphyseal region was higher in Nx2 and Nx6 compared to SO6 (Figure 3D). There were no differences between proportions of Dickkopf-1 and sclerostin-positive osteocytes in SO6 vs. SO2 (Figure 3C,D).

### 2.4. Correlation Analysis

According to Spearmen’s analysis, bone indices related to bone formation and gene expressions had statistically significant negative correlations with serum creatinine (Table 2). There were no correlations with FGF23 or PTH levels, except for *Xpr1* (Table 2). Bone resorption parameters (osteoclast number and eroded perimeter) were negatively associated with serum phosphate level (Table 2).

## 3. Discussion

The study focused on bone changes at earlier stages of experimental CKD. In particular, compared to controls (SO2), sham-operated SHR (SO6) had features of CKD manifesting as albuminuria and a decreased serum Klotho level. Nephrectomized animals (Nx2 and Nx6) additionally had a significant change in serum creatinine, CCr, and IF, on average not exceeding 30% vs. controls (Table 1). Thus, kidney injury in applied experimental models approximately corresponded to stage 1 (SO6) or stage 2 (Nx2, Nx6) of human CKD.

Taking into consideration the evidence of bone remodeling in more advanced CKD [2,6,8,13,14,15,16,17], here we found that even mild kidney dysfunction is also associated with a bone response. First, in all applied experimental CKD models (SO6, Nx2, Nx6), we observed a decline in the integral indexes of bone exchange, living osteocyte population, and trabecular bone area. The ability of osteocytes to regulate osteoblast and osteoclast function (through cWnt and nuclear factor-ƙB ligand (RANKL)/osteoprotegerin (OPG)–mediated mechanisms) suggests the principal role of the osteocyte network in coordinating bone remodeling [9,22,23]. Hence, a lower osteocyte number and trabecular bone area, indicating a balance between bone formation and bone resorption, could represent features of initial bone turnover decline as a response to kidney dysfunction. Second, in nephrectomy models with a higher extent of chronic kidney injury compared to SO6, a decline in active osteoblasts, a bone formation index (in Nx2 and Nx6), and eroded perimeter, a resorption index (in Nx6), likely mirrored further steps of CKD-MBD progression toward a low bone turnover phenotype [8,12,24]. In the SO6 and Nx2 CKD models, trabecular bone area, osteocytes (in SO6 and Nx2), and osteoblasts (in Nx2) decreased without changes in osteoclasts or eroded perimeter. These data allow suggesting that decline in bone formation occurs early in the course of CKD, further accompanied by bone resorption lowering in Nx6 as evaluated by eroded perimeter (see Figure 1).

Overt CKD-MBD comprises alterations in serum phosphate, PTH, and FGF23 that are crucial and tightly interconnected factors of bone turnover regulation [2,9]. Unlike other studies, our experimental approach allowed assessing a bone response to CKD before the development of hyperphosphatemia and systemic elevation of conventional phosphate regulatory factors, FGF23 and PTH [6,7,8,11,12,13,15,20,21,25]. In our experimental CKD models with unchanged serum PTH and FGF23 levels, histological bone alterations seemed to increase concurrently with the extent of kidney injury (see Figure 1). It was also obvious in the pooled correlation analysis, where bone indices were mostly associated with serum creatinine but not with PTH and FGF23 levels (see Table 2). Consistent with our data, osteoblast dysfunction and reduced bone formation progressed in parallel to the extent of kidney dysfunction at normal PTH levels [12]. These data raise a question about the importance of other CKD-induced mechanisms of bone turnover alterations apart from Pi/PTH/FGF23 axis up-regulation.

One could suggest that the initial predominance of inhibitory conditions in bone cells is a way toward a decline in bone turnover. These conditions may include PTH suppression and/or bone resistance to the PTH action, repression of osteocyte cWnt signaling, and increased expression of iWnt such as sclerostin, Dickkopf-1, and secreted frizzled-related protein 4 [6,8,12,13].

A limited number of prior studies have addressed these issues. PTH/PTHr receptor signaling is associated with increased bone turnover by cWnt up-regulation [26] and a decrease in iWnt [26,27]. Studies involving advanced experimental and human CKD revealed a decrease in PTH/PTHrP receptor [28,29]. Importantly, that reduced bone formation in mild chronic kidney injury was associated with a downregulation of the PTH/PTHrP receptor [12]. Subsequently, reduced PTH/PTHr receptor signaling could cause bone PTH resistance at the normal systemic PTH level [12]. 

Extracellular Pi regulates cWnt by overexpressing iWnt [8,13,30]. Hence, hyperphosphatemia and Pi loading could aggravate low bone turnover in more advanced CKD [8,9,30]. Consistently with these findings, we observed an increase in sclerostin and Dickkopf-1 serum levels along with serum Pi elevation and a decline in the histological indices of bone formation and resorption in Nx6 animals. Likewise, serum Pi negatively correlated with osteoclast-related bone indices (see Table 2) in a pooled SHR group.

To capture the additional early molecular events in the progression of CKD-MBD, we examined closely interrelated pathways associated with bone remodeling at the gene expression level. Studied gene products are involved in all major biological processes of bone regulation, including osteoblasts and osteoclasts differentiation, bone mineralization, ossification, phosphate homeostasis, ion transport, and extracellular matrix organization (see Figure S4). Hence, a down-regulation of these genes may be the molecular basis of the morphological features of low bone turnover detected in mild CKD models and characterized by the decline in the number of osteoblasts, osteocytes, trabecular bone area, and eroded perimeter. In parallel with the bone histomorphometric alterations in Nx2 and Nx6, we found the down-regulation of gene expression profiles directly related to osteoblastogenesis (*Sp7*, *Ctnnb1*, and *Bmp4)* [31,32,33]. Decreased expression of genes regulating Pi transport and sensing as well as osteoblast differentiation was also apparent (*Slc20a1*, *Slc20a2*, *Xpr1*, *Ankh*, *Fgfr2*, *Mapk1*, *Mapk3*) [34,35]. Lower osteoblast number and bone area are likely related to the decline in the osteoblast-regulatory gene profile in Nx2 and Nx6 groups compared to SO6. Contrariwise, there were no differences in osteoclast regulatory gene expression (*Tnfrsf11B*, *Tnfsf11*, and *Lgr4* [9]) between experimental groups (SO6, Nx2, and Nx6) and control (SO2), consistent with no histological alterations in osteoclast number.

The studied gene expression profile remained unchanged compared to controls in the earliest CKD model, SO6, associated with albuminuria and serum Klotho decline only. In this model, we observed osteocyte depopulation without a decrease in osteoblasts, the precursors of early osteocytes. This finding likely emphasizes a disorder of osteoblast maturation or increased mature osteocyte apoptosis [8,22,23], possibly mediated by PTH/PTHrP receptor down-regulation [12,26,27,28,29] and lower bone DMP1 expression in advanced CKD-MBD and overt hyperphosphatemia [23,36]. In much earlier experimental CKD-MBD stages, we did not observe a reduced bone *Dmp1* expression, while other potential mechanisms of the osteocyte regulation at early CKD require further investigation due to the current lack of relevant studies.

Prior studies revealed down-regulation of *Ctnnb1* (β-catenin), representing the cWnt repression mediated by iWnt as a mechanism of osteoblastogenesis alterations [6]. However, we found that in Nx2 and Nx6, a decline in *Ctnnb1* mRNA occurred in the absence of a significant elevation in bone expression of iWnt genes, *Dkk1*, *Sost*, and *Sfrp2*. In parallel, there was an increase in proportions of Dickkopf-1 and sclerostin-positive osteocytes in nephrectomy CKD groups, likely representing a non-genomic mechanism of bone cWnt down-regulation. These findings could also suggest the role of post-translational modification and stability in the cytoplasm in the maintenance of these proteins’ levels and iWnt inhibitory functions rather than gene expression regulation. In addition, the down-regulation in genes of osteoblast pathways, cWnt (*Ctnnb1*) and MAPK (*Mapk1/3*), might also be mediated by a significant decline in *Fgfr2* and *Bmp4* (Table S2, Figure 2 and Figure S4), consistent with prior studies [30,37].

The Pi transport systems (*Slc20a1*, *Slc20a2*, *Xpr1*, and *Ankh*) are important for either the maintenance of bone and systemic Pi exchange or for osteoblasts and osteoclasts differentiation, bone resorption, and matrix mineralization [38,39,40,41] (Figure S4). According to our data, the downregulation in these genes and their eventual signaling (*Fgfr2*, *Mapk1/3*, *Bmp4*, and *Ctnnb1*) appears to be an early bone response to mild CKD, concurrently with histological features of lower bone turnover in Nx2 and Nx6 (vs. SO6 and SO2).

Phosphate transporters, particularly PiT-1 and PiT-2, are regulated by ambient Pi, likely serving Pi sensing and controlling the phosphate cell content and transcription [34,35]. The effects of extracellular Pi on the expression of genes related to its transport and or sensing are dose- and time-dependent [30,42]. Short-term responses to supraphysiological concentrations of Pi or hydroxyapatite exposure in cell media led to up-regulation in genes coding for Pi transporters (*Slc20a1*, *Slc20a2*) and downstream MAPK signaling molecules [34,41]. Contrariwise, the impact of the lower Pi concentrations is associated with *Slc20a2* down-regulation in vitro [43]. Phosphate renal retention is associated with a decline in cWnt ligands/receptors and iWnt up-regulation [8].

In Nx6 animals that exhibited hyperphosphatemia, we found *Slc20a1/2*, *Xpr1*, and *Mapk1/3* to be downregulated, suggesting opposite effects of longer bone exposure on increased extracellular Pi in the regulation of gene expression in in vitro vs. short-term in vitro studies. In the Nx2 CKD model, a similar pattern of gene expression was obvious, however, without serum Pi elevation. Since it was found that Pi-transporters are regulated by extracellular Pi [34,35], one could hypothesize that postprandial serum Pi load and transient local bone Pi distribution are involved in transcription regulation, even in fasting normophosphatemia. This hypothesis, however, requires further study with an appropriate design.

The simultaneous down-regulation of Pi transport and export systems seems to prevent bone Pi overload, as the total bone phosphorus content was unchanged in mild CKD models. Subsequently, a decrease in trabecular bone and the Pi-buffering capacity of the skeleton in settings of CKD-induced Pi imbalance could predispose to further re-distribution of this anion toward the cardiovascular system [44].

Overall, our findings are consistent with the clinical evidence that, in earlier CKD stages, adynamic bone disease characterized by low bone turnover occurs in a significant proportion of patients [18,19,20]. The observed bone response even in mild experimental CKD may be of importance for planning and conducting explorative studies of earlier human CKD stages and for the further development of strategies aiming to prevent the overt, mainly irreversible clinical consequences of CKD-MBD.

### Research Limitations 

Our research has several limitations. First, we did not perform dynamic histomorphometry of bone and mineralization indexes. However, in this exploratory study, we found the difference between CKD groups and controls using histomorphometric assessment of static bone parameters and gene expression profiles that are tightly related to bone turnover, as indicated earlier [8,9,12,32,33,34,35,36,37,38,39]. Next, serum Pi levels, its excretion, and regulatory factors were assessed only in the fasting state. Thus, we were not able to evaluate whether diurnal variations of Pi and its postprandial load in CKD have an impact on bone histology and gene expression profiles. Additionally, to elucidate the role of intestinal Pi load, it seemed feasible to include groups fed a high phosphate diet. However, in planning this study, we intentionally did not include such groups to avoid hyperphosphatemia and secondary hyperparathyroidism, which exert well-known effects on bone turnover [2,9].

## 4. Materials and Methods

### 4.1. Animals

Animals were obtained from the Pavlov Institute of Physiology (Saint Petersburg, Russia). The study was conducted according to the Code of Practice for the Housing and Care of Animals Used in Scientific Procedures and approved by the local Ethics Committee of Pavlov First Saint Petersburg State Medical University (animal ethics approval code No. 02-2013, protocol code No. 206, April 23, 2018) and adhered to the European Community Council Directive (2010/63EU) and the guidelines of the National Institute of Health (Guide for the Care and Use of Laboratory Animals).

Adult male spontaneously hypertensive rats (SHR) and Wistar Kyoto (WKY) rats weighting 190–230 g were housed using a 12-h/12-h daylight cycle at room temperature (20–22 °C) with ad libitum access to water and standard rat chow containing 0.6% phosphate. 

We induced mild CKD in SHR by arterial hypertension (AH) exposure combined with a sham operation (SO) or 3/4 nephrectomy (Nx) (Table 1, Figure S1). SHR with two-month AH exposure served as controls (SO2). We obtained three groups of experimental mild CKD: (i) AH-induced in SO SHRs with six-month exposure (SO6); (ii) AH and Nx with two-month exposure (Nx2); and (iii) AH and Nx with six-month exposure (Nx6). The hypertension is a kidney injury factor in SHRs, and we also used a sham Wistar Kyoto with a 2-month follow-up (WKY2) as a normotensive control. The only purpose for using the WKY2 group was to confirm that the SO2 group had normal kidney function and phosphate metabolism (compared to WKY2). Since Wistar Kyoto and SHR are genetically different strains [45], we did not use the WKY2 group to compare gene expression and bone metabolism indices to SHRs. Between SHR groups, we made the following comparisons: control SO2 vs. all mild CKD groups (SO6, Nx2, Nx6); earlier stages of mild CKD (SO6) vs. Nx2 and Nx6; and Nx2 vs. Nx6.

Systolic blood pressure was measured the day before the euthanasia via the tail-cuff method using an electrometer (ELEMA, Lund, Sweden) and registered at a paper speed of 10 mm/s. Blood, left-side kidney, and tibia samples were harvested immediately after sacrifice. 24-h urine samples were collected the day before.

### 4.2. Laboratory Measurements

The blood and 24-h urine samples were centrifuged at 3000 rpm for 10 min, aliquoted, and stored at −80 °C with temperature control. The stored samples underwent a single thaw followed by assays. The levels of creatinine (by the enzymatic method), Pi, and urea were measured using reagent kits on SYNCHRON CX DELTA (Beckman Coulter, Brea, CA, USA). The levels of urinary albumin were measured by immunoturbidimetry using reagent kits (Vital, Saint Petersburg, Russia) on an analyzer CA-90 (Furuno, Nagasaki, Japan). The levels of intact PTH and intact FGF23, dickkopf-1, sclerostin were measured using a MILLIPLEX MAP «Rat Bone Magnetic Bead Panel 1» (EDM Millipore Corporation, Billerica, MA, USA) on Bio-Plex 200 Reader (BioRad, Hercules, CA, USA), and serum α-Klotho—using ELISA Kit for Rat (Cloud-Clone Corp., Katy, TX, USA) on Microplate Reader Immunochem 2100 (High Technology, North Attleborough, MA, USA).

### 4.3. Inductively Coupled Plasma Atomic Emission Spectroscopy

The tibial diaphysis and kidney were sampled and stored at −80 °C with temperature control. An inductively coupled plasma-atomic emission spectrometry method was used for the measurement of phosphorus in bones and kidneys. The specimens were mineralized by nitric acid (Merck, Darmstadt, Germany) with subsequent microwave decomposition: a temperature-time ramp for 20 min with a final temperature of 210 °C, then a 25-min hold time at 1500 W at 210 °C. The analysis was performed with an ICPE-9000 (Shimadzu, Kyoto, Japan) with the following parameters: radio frequency power 1550 W, sample depth 10 mm, carrier gas 0.65 L/min, nebulizer pump 0.10 rps, spray chamber temperature 13 °C (55.4 °F), and dilution gas 0.40 L/min as described previously [46].

### 4.4. Real-Time Polymerase Chain Reaction

Tibial diaphyses were flushed with phosphate-buffered saline to remove all the bone marrow and were incubated overnight at 4 °C with RNAlater solution (Evrogen, Moscow, Russia) and then stored at −80 °C with temperature control. Then bone samples were ground to a fine powder using a mortar and pestle under liquid nitrogen in RNase-free conditions. Total RNA was extracted using the TriZ reagent RNA Kit (Inogene, Saint Petersburg, Russia) following the manufacturer’s instructions. The extracted RNA was eluted in RNAse-free water. A reverse transcriptase reaction was performed with the RevertAid First Strand cDNA Synthesis Kit (Thermo Scientific, Waltham, MA, USA). For each generated cDNA sample, multiplex qPCR was performed for genes of interest and glyceraldehyde-3-phosphate-dehydrogenase (Table S1). All reactions were adapted from the manufacturer’s protocol (Syntol, Moscow, Russia, M-428), containing 2.5 mM of each dNTP, x10PCR buffer, 5 units of Taq-DNA polymerase, and 2.5 µL of 25 mM MgCl_2_, supplemented with 7 pmol of each gene-specific primer, 5 pmol of Taqman probes for the genes of interest, and glyceraldehyde-3-phosphate-dehydrogenase. The final reaction volume was 25 µL. Quantitative real-time PCR was performed with the BioRad CFX 96 (BioRad, USA). The amplification protocol was 95 °C for 10 min, followed by 45 cycles of heating at 95 °C for 15 s, annealing at 60 °C for 1 min, and signal detection. The relative expression gene of interest level was calculated using the Delta Ct method and expressed as a percent value. All PCR results for one gene in one animal represented mean values of triplicated measurements for each mRNA level. Studied genes, including differentially expressed ones, were further analyzed with the use of the Search Tool for Recurring Instances of Neighboring Genes Database to predict interaction networks and the biological processes in which they are involved “https://string-db.org/ (accessed on 15 March 2023)”.

### 4.5. Histology and Immunohistochemistry

Two-mm midcoronal renal slices and tibial distal metaphysis with diaphysis were fixed in buffered 4% formaldehyde for 24 h and 48 h, respectively. Afterward, tibial samples were washed in distilled water, followed by 10% EDTA (pH 7.4) incubation for around two months (needle test). The EDTA solution was replaced twice a week. Processed tissues were embedded in paraffin and cut into two-micron sections, dewaxed, rehydrated, and stained.

For bone IHC, after retrieval with Proteinase K for 20 min at 37 °C and endogen peroxidase blocking, sections were incubated with rabbit polyclonal antibodies to Dickkopf-1 (1:400 dilution, ab109416, Abcam, Cambridge, UK) and sclerostin (1:100 dilution, ab63097, Abcam, UK) overnight at 4 °C, followed by an anti-rabbit Histofine^®^ Simple StainTM MAX PO I detection system for 30 min at room temperature (Nichirei Biosciences, Inc., Tokyo, Japan). A 3,3-diaminobenzidine Histofine^®^ DAB-3S kit (Nichirei Biosciences Inc., Tokyo, Japan) was used as the chromogen. Finally, the slides were counterstained with hematoxylin and mounted after dehydration.

### 4.6. Quantitative Morphometry

Renal IF area, static bone histomorphometric indices, and IHC-positive osteocytes were calculated quantitatively with the two examiners blinded to the study groups who examined ten fields of view (400× magnification, 10×/22) for a section or in a whole slide image using the freeware Orbit Image Analysis Version 3.64 and Pannoramic Viewer 1.15.4.

IF was measured at the renal cortex in the areas without glomeruli in ten random fields of view for the slide and expressed as a percent of Masson’s trichrome-stained blue color area using Orbit Image Analysis. The mean values for each animal were analyzed afterward.

Bone histomorphometric parameters were measured in compliance with «Bone research protocols, Methods in molecular biology (Histomorphometry in Rodents)» [47] and the recommendations of the nomenclature committee of the American Society for Bone and Mineral Research [48].

To quantify the bone area in the metaphyseal region, the trabecular bone area and bone tissue area were measured in three whole slide images for sections spaced at least 100 µm from each other for one animal. Trabecular bone area was standardized to bone tissue area (B.Ar%T.Ar), and the mean values of three measurements for each animal were analyzed (N = 96 [32 animals × 3 whole slide images], n = 8 for each group).

The number of active osteoblasts (N.Ob) and osteoclasts (N.Oc), the eroded perimeter (E.Pm) and bone perimeter (B.Pm) in the metaphyseal region, and the number of osteocytes (N.Ot) in the diaphyseal region were calculated in 8–10 fields of view for each animal. N.Ot were standardized to the bone tissue area. N.Ob, N.Oc, and E.Pm were standardized to B.Pm. Values for individual fields of view (72–80 per group) were further used for statistical analyses. 

To quantify the bone expression of Dickkopf-1 and sclerostin, IHC-staining was classified as “present” or “absent” in any given osteocyte, and the total number of osteocytes with positive staining was counted and normalized to all osteocytes in five fields of view in the diaphyseal region for section using Orbit Image Analysis (n = 40 for each group for each parameter). These normalized values for each field of view were used for further statistical tests.

### 4.7. Statistical Analyses

Analyses were performed using SAS version 9.4 (SAS Institute Inc., Cary, NC, USA). Values are expressed as medians [interquartile range (IQR)]. Groups were compared using a two-tailed Mann-Whitney U-test and a Kruskal-Wallis H-test. The association between variables was evaluated by Spearman’s coefficient. Statistical significance was defined as *p*-values < 0.05.

## 5. Conclusions

In conclusion, the bone response to mild experimental CKD is associated with histological and molecular alterations that are suggestive of lower bone turnover. These findings could have relevance to the clinical setting of CKD-MBD and are particularly important for planning further experimental and human research in this area, focusing on pathophysiology, early recognition, and prevention of the disease’s progression.

## Figures and Tables

**Table 1:** Parameters studied in sham-operated and nephrectomized rats.

Name	WKY2	SO2	SO6	Nx2	Nx6
Group number	(1)	(2)	(3)	(4)	(5)
Strain	Wistar Kyoto rats	Spontaneously hypertensive rats			
Model	normotensive control	control	mild CKD models		
Surgery	sham-operated	sham-operated	sham-operated	3/4 nephrectomy	3/4 nephrectomy
Duration of the experiment, mo	2	2	6	2	6
Rats number, n	8	8	8	8	8
Initial body weight, g	228 (224;230)	220 (215;226)	215 (207;228)	224 (217;228)	222 (212;229)
Final body weight, g	345 (336;361)	317 (311;337)	317 (306;336)	320 (300;370)	331 (309;365)
Systolic blood pressure, mmHg	135 (130;142) ^2–4#^	170 (160;182) ^3,4*5#^	195 (183;200)	195 (180;205)	208 (195;223)
Serum creatinine, μmol/L	74 (69;79) ^3–5#^	73 (68;77) ^3–5‡^	83 (81;86) ^4,5#^	93 (91;97) ^5#^	107 (102;110)
Urea, mmol/L	4.89 (3.81;6.93) ^3–5#^	5.36 (4.19;6.41) ^4,5†^	5.37 (4.36;7.09) ^4,5#^	7.10 (6.95;7.58) ^5#^	10.7 (9.63;12.4)
Creatinine clearance, mL/min/100 g	0.20 (0.15;0.26)	0.27 (0.20;0.35^) 4,5‡^	0.23 (0.14;0.30)	0.19 (0.16;0.23)	0.19 (0.16;0.25)
Urinary albumin/creatinine, mg/mg	0.026 (0.017;0.035) ^3–5#^	0.043 (0.031;0.065) ^3–5‡^	0.288 (0.237;0.336)	0.327 (0.153–0.370)	0.543 (0.345;1.114)
Renal interstitial fibrosis, %	2.5 (1.6;3.1) ^3–5#^	1.9 (0.1;3.3) ^3–5#^	5.8 (3.5;7.2) ^5#^	6.9 (3.9;7.7) ^5#^	14.5 (13.2;17.2)
Serum Klotho, pg/mL	2698 (2413;2831)	2916 (2520;5374) ^3–5^*	2043 (1676;2663)	2304 (2074;2524)	2259 (1428;2696)
Serum inorganic phosphate, mmol/L	1.47 (1.22;1.60) ^3–5#^	1.89 (1.79;1.95) ^5^*	1.90 (1.80;1.98) ^5‡^	1.60 (1.50;1.84) ^5^*	2.21 (2.15;2.28)
Urinary phosphate/creatinine, mg/mg	5.6 (4.5;6.5) ^5^*	8.9 (6.9;10.1)	8.6 (7.9;9.8)	10.1 (7.6;12.7)	9.3 (8.9;11.2)
Bone phosphorus, g/kg	58.6 (33.4;62.7)	63.5 (58.1;64.5)	62.8 (61.8;64.1)	62.8 (55.2;65.6)	59.7 (58.9;63.6)
Kidney phosphorus, mg/kg	818 (770;877)	872 (606;1241)	822 (637;1024)	699 (668;825)	734 (671;862)
Intact parathyroid hormone, pg/mL	55.1(12.7;112.9)	76.6 (18.4;111.0)	45.5 (12.6;67.1)	45.9 (21.2;76.6)	33.5 (9.6;84.9)
Intact fibroblast growth factor 23, pg/mL	351 (290;836)	361 (330;1530)	468 (326;694)	676 (330;793)	630 (330;953)
Serum dickkopf-1, pg/mL	965 (845;1175)	1221(975;1534)	466(100;979) ^4*5†^	1402 (994;1605)	1017 (876;1264)
Serum sclerostin, pg/mL	233 (161;292)	246 (157;433)	94 (50;169) ^5*^	212 (119;251)	221 (161;263)

Superscripts correspond to *p*-values of inter-group differences (each group is indicated by a group number); * *p* < 0.05, † *p* < 0.01, ‡ *p* < 0.005, # *p* < 0.001.

**Table 2:** Regression analysis of the association between serum creatinine, phosphate indices, bone histology, and gene expression in a pooled SHR group.

	PTH, pg/mL	FGF23, pg/mL	Serum Pi, mmol/L	Urinary Pi/Cr, mg/mg	Serum Cr, mmol/L
N.Ot/T.Ar, no/mm^2^	0.11	0.20	−0.10	0.16	−0.31 *
N.Ob/B.Pm, no/mm	−0.38	−0.22	−0.10	−0.26	−0.32 *
N.Oc/B.Pm, no/mm	−0.16	−0.11	−0.37 *	−0.11	0.06
E.Pm.%B.Pm	−0.12	0.09	−0.48 *	−0.10	−0.22
*Slc20a1*	−0.05	0.05	0.29	−0.24	−0.39 *
*Slc20a2*	−0.14	0.04	0.16	−0.37 *	−0.43 *
*Xpr1*	−0.54 *	−0.004	−0.03	−0.16	−0.50 *
*Ankh*	−0.26	0.03	0.14	−0.16	−0.40 *
*Fgf23*	−0.07	−0.18	0.06	0.04	0.45 *
*Fgfr2*	−0.08	−0.06	0.04	−0.03	−0.37 *
*Mapk1*	0.01	0.20	0.24	−0.30	−0.57 **
*Mapk3*	−0.28	−0.07	−0.13	−0.26	−0.50 *
*Sp7*	0.12	0.15	0.03	0.02	−0.44 *
*Ctnnb1*	−0.17	0.05	−0.08	−0.15	−0.43 *
*Wnt10b*	−0.06	−0.08	0.01	−0.08	−0.34 *
*Vdr*	−0.02	0.02	0.06	0.04	−0.34 *
*Bmp4*	0.12	0.24	−0.13	−0.03	−0.42 *

N.Ot/T.Ar—osteocyte number, N.Ob/B.Pm—osteoblast number, N.Oc/B.Pm—osteoclast number, E.Pm.%B.Pm—eroded perimeter, *Slc20a1*—solute carrier family 20 member 1 (Pit-1); *Slc20a2*—solute carrier family 20 member 2 (Pit2), *Xpr1*—xenotropic and polytropic retrovirus receptor 1, *Ankh*—ANKH pyrophosphate transport regulator, *Fgf23*—fibroblast growth factor 23, *Fgfr2*—fibroblast growth factor receptor 2, *Mapk1*—mitogen-activated protein kinase 1 (Erk2), *Mapk3*—mitogen-activated protein kinase 3 (Erk1), *Sp7*—transcription factor Osterix, *Ctnnb1*—catenin beta 1, *Wnt10b*—Wnt family member 10B, *Vdr*—vitamin D receptor, *Bmp4*—bone morphogenetic protein 4, *—indicates significant correlation at *p* < 0.05, **—indicates significant correlation at *p* < 0.005.

## Data Availability

All data presented in this study are available from the corresponding author on reasonable request.

## Supplementary Materials

The following supporting information can be downloaded at: https://www.mdpi.com/article/10.3390/ijms24087270/s1.
