# Supplementary material for: Mild Chronic Kidney Disease Associated with Low Bone Formation and Decrease in Phosphate Transporters and Signaling Pathways Gene Expression
Source: Int J Mol Sci. 2023 Apr 14;24(8):7270. doi: 10.3390/ijms24087270 (PMC10138582; doi:10.3390/ijms24087270)
Supplement: Supplementary file 1 [file ijms-24-07270-s001.zip › Figure S1.pdf]

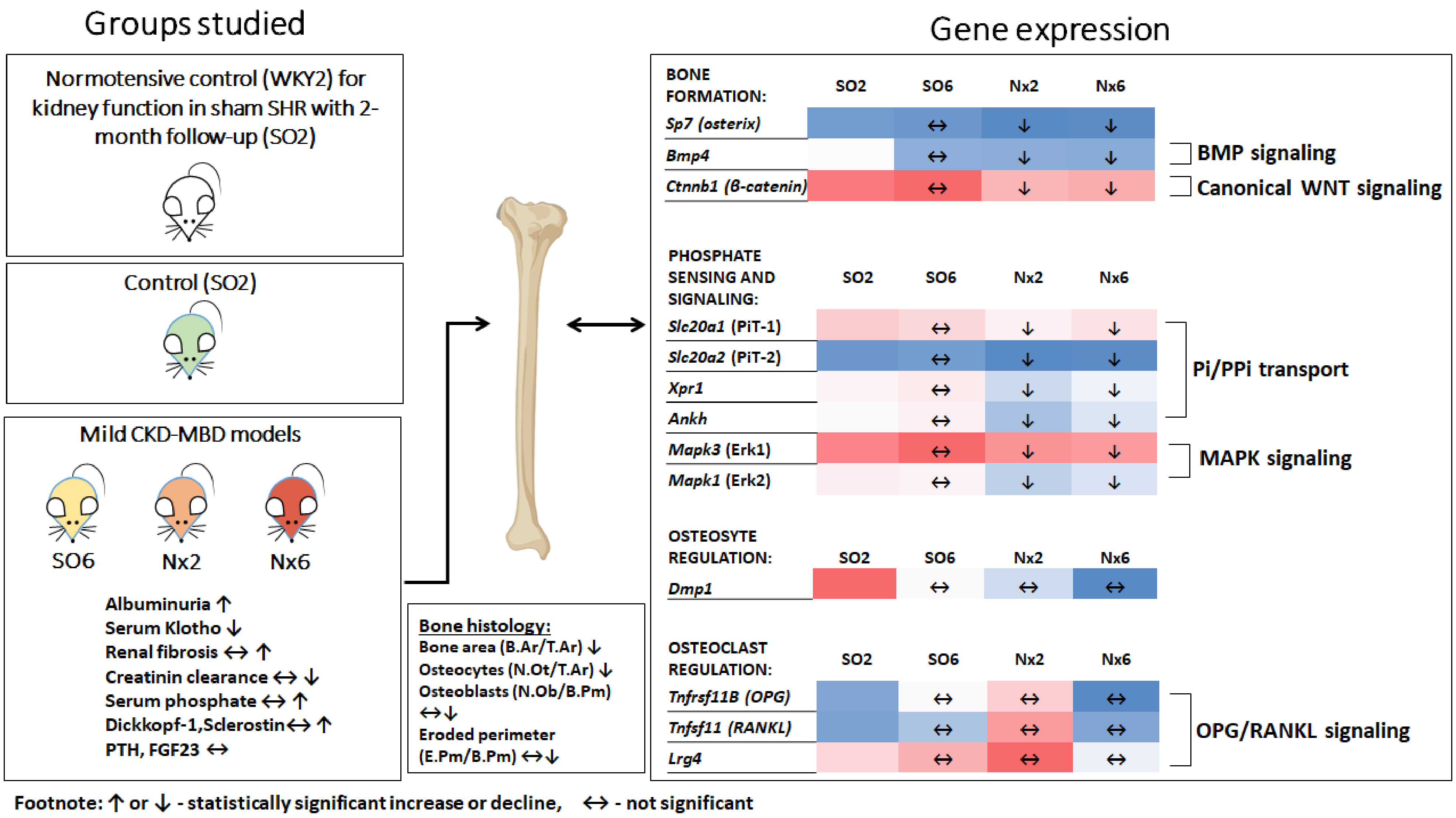

Figure S1. Mild CKD-MBD models, bone remodeling and related pathways at the level of gene expression: WKY2 – sham Wistar Kyoto rats with 2-month follow-up, SO2 – sham SHR with 2-month follow-up, SO6 – sham SHR with 6-month follow-up, Nx2 – 3/4 nephrectomy in SHR with 2-month follow-up, Nx6 – 3/4 nephrectomy in SHR with 6-month follow-up; Sp7 – Sp7 transcription factor (osterix); Bmp4 – bone morphogenetic protein 4; Ctnnb1 – catenin beta 1; Slc20a1 – solute carrier family 20 member 1 (Pit-1); Slc20a2 – solute carrier family 20 member 2 (Pit2); Xpr1 – xenotropic and polytropic retrovirus receptor 1; Ankh – ANKH PPi transport regulator; Mapk3 – mitogen activated protein kinase 3 (Erk1); Mapk1 – mitogen activated protein kinase 1 (Erk2); Dmp1 – dentin matrix acidic phosphoprotein 1; Tnfrsf11B – TNF receptor superfamily member 11 B (OPG); Tnfsf11 – TNF superfamily member 11 (RANKL); Lgr4 – leucine-rich repeat-containing G protein-coupled receptor 4; Pi – inorganic phosphate; PPi – pyrophosphate; OPG – osteoprotegerin; RANKL – receptor activator of nuclear factor kappa-B ligand.
