# Supplementary material for: Mild Chronic Kidney Disease Associated with Low Bone Formation and Decrease in Phosphate Transporters and Signaling Pathways Gene Expression
Source: Int J Mol Sci. 2023 Apr 14;24(8):7270. doi: 10.3390/ijms24087270 (PMC10138582; doi:10.3390/ijms24087270)
Supplement: Supplementary file 1 [file ijms-24-07270-s001.zip › Figure S3.pdf]

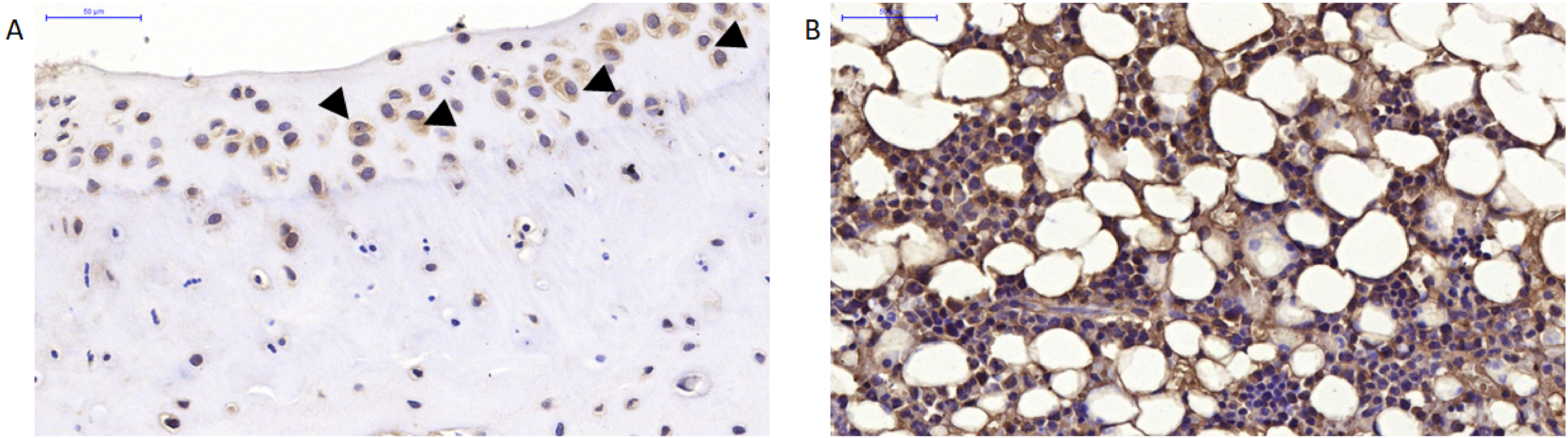

Figure S3. Representative microphotographs for sclerostin IHC-staining in chondroblasts (A, arrowheads), and bone marrow (B).
