# Supplementary material for: Mild Chronic Kidney Disease Associated with Low Bone Formation and Decrease in Phosphate Transporters and Signaling Pathways Gene Expression
Source: Int J Mol Sci. 2023 Apr 14;24(8):7270. doi: 10.3390/ijms24087270 (PMC10138582; doi:10.3390/ijms24087270)
Supplement: Supplementary file 1 [file ijms-24-07270-s001.zip › Table S1.pdf]

Table S1. Primer and probe sequences for RT-PCR

Gene	Primer sequence (5'→3')	Probe R-sequence–Q (5'→3')
Phosphate and pyrophosphate transporters		
Slc20a1	F: CTCATCCTGGGCTTCATCAT	FAM-CATTTGTCTTGGCATTCTCCGTGGG-BHQ1
	R: CCGGATGGTTTCACTCACTT	
Slc20a2	F: GCTCTACCATTGGCTTCTCG	R6G-ATCGTTGCCTCCTGGTTCATATCCC-BHQ1
	R: ACAGAGGAAGTGCCTGGAGA	
Xpr1	F: AAGACGTGATTCTGCGCTTT	ROX-TGCTACAACTTTTAAGCCTCATGTTGGG-BHQ2
	R: CACGGAATTCACCACAGTTG	
Ankh	F: CAAGAGAGACAGGGCCAAAG	FAM-CAGTCTTCCACACCCTGATAGCCTBHQ1
	R: AAGGCAGCGAGATACAGGAA	
MAPK signaling		
Mapk3	F: TCCAAGGGCTACACCAAATC	FAM-CTACCTGGACCAGCTCAACCACA-BHQ1
	R: AGGTAGTTTCGGGCCTTCAT	
Mapk1	F: TTGCTGAAGCACCATTCAAG	R6G-CAGGACAAGGGCTCAGAGGACTG-BHQ1
	R: ACGGCTCAAAGGAGTCAAGA	
Klotho/FGF23 regulatory axis		
Kl	F: AGCTGCTTGTGTTGTGATGC	FAM-ATGGTGGCGGTTTTAAACAGGCA-BHQ1
	R: TACGGGGGTGCTGTAGAAAC	
Fgfr2	F: ACTGGACCAACACCGAAAAG	FAM-ACGAAACCAGCACTGGAGCCTTATT-BHQ1
	R: CTCCACCAGGCAGGTGTAAT	
Fgf23	F: TGGGCACTGCTAGAGCCTAT	ROX-CAAGGTGTACAGTGACCCCCAGC-BHQ2
	R: GCGGAGATCCATACAAAGGA	
Canonical Wnt signaling and its inhibitors		
Ctnnb1	F: GCCAGTGGATTCCGTACTGT	Cy5-CACCACGCTGCATAATCTCCTGCT-BHQ2
	R: GAGCTTGCTTTCCTGATTGC	
Wnt10b	F: GCACTGTCTAGGGGCAAGAG	Cy5-CCAGCCCTATTCCTGGCTCTGTC-BHQ2
	R: CACTTCCGCTTCAGGTTTTC	
Fzd2	F: GAACTCCTGCGCTACTCACC	ROX-CCTCAAGGTGCCGTCCTATCTCA-BHQ2
	R: TCCTCCTGCGAGAAGAACAT	
Dkk1	F: TTACTGTGGGGAAGGTCTGG	Cy5-CCAACAGCCTAAATGCGATGGACTC-BHQ2
	R: ACATCCTTGGGATTGAGCTG	
Sost	F: CAGCTCTCACTAGCCCCTTG	ROX-CTGCTTGTACATGCAGCCTTCGT-BHQ2
	R: CGGTTCATGGTCTGGTTGTT	
Sfrp2	F: TGTCCGATAGGGACCTGAAG	R6G-TGGGACAGAAACAGGGTGGAGAG-BHQ1
	R: CGAGAAGCCACTCCACTAGG	
VDR/OPG/RANKL regulatory axis		
Vdr	F: AACTCCTCCTCCTCCAGCTC	ROX-CCTGTCTCCTCTCTCCATGCTGCBHQ2
	R: CTGGTCATCGGAGGTGAGAT	
Cyp27b1	F: GGTGAGAGGCTTGGCTAGTG	Cy5-ATGGGGACAGTTGAAACTGCACCTT-BHQ2
	R: TCTGGAGTTCAGGAGCCAGT	
Tnfrsf11B	F: GAATGGTCACTGGGCTGTTT	Cy5-TGGGAATGAAGATCCTCCAGCCC-BHQ2
	R: CCTCTTTCTCAGGGTGCTTG	
Tnfsf11	F: CATCGGGTTCCCATAAAGTCAGT	FAM-TCAGGCATCATGAAACCTCAGGGAG-BHQ1
	R: GAACTTGGGATTTTGATGCTGGT	
Lgr4	F: GGGAAGAGCAGTCACCTCAG	R6G-CCCTCTTAGCTTTGCTGGGTGC-BHQ1
	R: TAACGATGGGGTTTCTCCTG	
Other osteogenesis genes		
Sp7	F: CACTGGCTCCTGGTTCTCTC	R6G-AGCTCACTATGGCTCCAGTCCC-BHQ1
	R: GGGGCTGAAAGGTCAGTGTA	
Bmp4	F: TAGGAGCCATTCCGTAGTGC	ROX- TCTCTGAGCCTTTCCAGCAAGTTTGT-BHQ2
	R: CTTCCCGGTCTCAGGTATCA	

Dmp1	F: CGGCTGGTGGTCTCTCTAAG	
	R: CATCACTGTGGTGGTCCTTG	R6G-CAGTCCAGTGAAGACAGCACGTCT-BHQ1
Reference gene		
Gapdh	F: AGACAGCCGCATCTTCTTGT	
	R: CTTGCCGTGGGTAGAGTCAT	R6G-TGCCAGCCTCGTCTCATAGACAAG-BHQ1

Slc20a1 – solute carrier family 20 member 1 (Pit-1), *Slc20a2* – solute carrier family 20 member 2 (Pit2), *Xpr1* – xenotropic and polytropic retrovirus receptor 1, *Ankh* – ANKH PPi transport regulator, *Mapk3* – mitogen activated protein kinase 3 (Erk1), *Mapk1* – mitogen activated protein kinase 1 (Erk2), *KL* – Klotho, *Fgf23* – fibroblast growth factor 23, *Fgfr2* – fibroblast growth factor receptor 2, *Ctnnb1* – catenin beta 1, *Sfrp2* – secreted frizzled-related protein 2, *Fzd2* – frizzled class receptor 2, *Wnt10b* – Wnt family member 10B, *Sost* – sclerostin, *Dkk1* – dickkopf 1, *Vdr* – vitamin D receptor, *Cyp27b1* – cytochrome P450, family 27, subfamily b, polypeptide 1 (1-alpha-(OH)ase), *Tnfrsf11B* – TNF receptor superfamily member 11 B (OPG), *Tnfsf11* – TNF superfamily member 11 (RANKL), *Lgr4* – leucine-rich repeat-containing G protein-coupled receptor 4, *Sp7* – Sp7 transcription factor (osterix), *Bmp4* – bone morphogenetic protein 4, *Dmp1* – dentin matrix acidic phosphoprotein 1, *Gapdh* – glyceraldehyde-3-phosphate dehydrogenase.
