# Supplementary material for: Mild Chronic Kidney Disease Associated with Low Bone Formation and Decrease in Phosphate Transporters and Signaling Pathways Gene Expression
Source: Int J Mol Sci. 2023 Apr 14;24(8):7270. doi: 10.3390/ijms24087270 (PMC10138582; doi:10.3390/ijms24087270)
Supplement: Supplementary file 1 [file ijms-24-07270-s001.zip › Table S2.pdf]

Table S2. Bone mRNA relative expressions (tibial diaphysis)

	SO2 (1)	SO6 (2)	Nx2 (3)	Nx6 (4)
Fgf23, fibroblast growth factor 23	0.16 (0.10;0.20)	0.24 (0.15;0.28)	0.19 (0.18;0.22)	0.19 (0.11;0.24)
Fgfr2, fibroblast growth factor receptor 2	0.63 (0.43;0.76)	0.81(0.74;0.91)$^{3,4\#}$	0.41 (0.30;0.61)	0.57 (0.35;0.60)
Dkk1, dickkopf-1	0.3 (0.2;2.7)	0.4 (0.3;1.7)	1.4 (0.1;4.7)	0.2 (0.1;1.6)
Sost, sclerostin	1.2 (1.1;3.8)	2.1 (0.7;4.2)	1.9 (0.4;4.4)	1.6 (0.7;2.2)
Sfrp2, secreted frizzled-related protein 2	1.41 (0.78;4.53)	1.35 (0.77;1.86)	2.16 (0.47;5.23)	0.50 (0.46;2.62)
Fzd2, frizzled class receptor 2	0.42 (0.31;11.35)	0.69 (0.43;1.56)	1.64 (0.14;11.96)	0.25 (0.23;3.84)
Wnt10b, wnt family member 10B	0.03 (0.01;0.04)	0.02 (0.02;0.03)	0.02 (0.01;0.04)	0.02 (0.01;0.03)
Kl, klotho	0.22 (0.13;2.57)	0.31 (0.19;1.77)	1.44 (0.05;3.76)	0.22 (0.11;1.92)
Dmp1, dentin matrix acidic phosphoprotein 1	4.07 (3.11;6.80)	2.38 (1.71;4.98)	2.39 (0.88;5.83)	2.25 (1.40;5.00)
Cyp27b1, cytochrome P450, family 27, subfamily b, polypeptide 1	0.22 (0.10;4.15)	0.32 (0.16;1.07)	1.24 (0.06;3.49)	0.21 (0.10;1.12)
Tnfrsf11B, TNF receptor superfamily member 11B	0.25 (0.18;2.78)	0.57 (0.23;1.62)	1.01 (0.06;3.86)	0.16 (0.14;2.03)
Tnfsf11, TNF superfamily member 11	0.24 (0.11;2.49)	0.35 (0.21;1.38)	1.50 (0.10;4.42)	0.25 (0.13;1.58)
Lrg4, leucine-rich repeat-containing G protein-coupled receptor 4	0.93 (0.64;6.06)	1.32 (0.89;2.61)	2.01 (0.30;8.04)	0.50 (0.43;2.94)

Superscripts show p-values vs groups; #p<0.001.
